# Differences in Reporting of Violence and Deliberate Self Harm Related Injuries to Health and Police Authorities, Rawalpindi, Pakistan

**DOI:** 10.1371/journal.pone.0009373

**Published:** 2010-02-23

**Authors:** Umar Farooq, Mudassir Majeed, Junaid Ahmad Bhatti, Jahangir Sarwar Khan, Junaid Abdul Razzak, Muhammad Mussadiq Khan

**Affiliations:** 1 Surgical Unit I, Department of Surgery, Holy Family Hospital, Rawalpindi, Pakistan; 2 Equipe «Prévention et Prise en Charge des Traumatismes», Institut National de la Santé et de la Recherche Médicale Unité 897, Université Victor Segalen Bordeaux 2, Bordeaux, France; 3 Department of Emergency Medicine, Aga Khan University, Karachi, Pakistan; Aga Khan University Hospital, Pakistan

## Abstract

**Background:**

The aim of study was to assess differences in reporting of violence and deliberate self harm (DSH) related injuries to police and emergency department (ED) in an urban town of Pakistan.

**Methods/Principal Findings:**

Study setting was Rawalpindi city of 1.6 million inhabitants. Incidences of violence and DSH related injuries and deaths were estimated from record linkage of police and ED data. These were then compared to reported figures in both datasets. All persons reporting violence and DSH related injury to the police station, the public hospital's ED, or both in Rawalpindi city from July 1, 2007 to June 30, 2008 were included. In Rawalpindi city, 1 016 intentional injury victims reported to police whereas 3 012 reported to ED. Comparing violence related fatality estimates (N = 56, 95% CI: 46–64), police reported 75.0% and ED reported 42.8% of them. Comparing violence related injury estimates (N = 7 990, 95% CI: 7 322–8 565), police reported 12.1% and ED reported 33.2% of them. Comparing DSH related fatality estimates (N = 17, 95% CI: 4–30), police reported 17.7% and ED reported 47.1% of them. Comparing DSH related injury estimates (N = 809, 95% CI: 101–1 516), police reported 0.5% and ED reported 39.9% of them.

**Conclusion:**

In Rawalpindi city, police records were more likely to be complete for violence related deaths as compared to injuries due to same mechanism. As compared to ED, police reported DSH related injuries and deaths far less than those due to other types of violence.

## Introduction

Nearly one third of all injuries are intentional, half of which are self-inflicted whereas the other half result from interpersonal and collective violence [1], [2], [3]. In 2002, these injuries resulted in over 1.6 million deaths worldwide with an age adjusted mortality rate of 28.8 deaths per 100 000 inhabitants [2]. Interestingly, this disease burden was distributed differently between high-income (HICs) and low- and middle-income countries (LMICs) as more than 90% of these deaths occurred in LMICs [2], [3]. Mortality rate due to these injuries was twice as high in LMICs as compared to HICs (32.2 versus 14.4 per 100 000 inhabitants) [4].

Official crime statistics were the most common source of information used to assess intentional injury disease burden worldwide [4], [5], [6], [7]. Use of these statistics to compute injury rates was often criticized due to underreporting [4], [6]. Indeed, most of these injuries were never reported to the law enforcement agencies. A South African study showed that only 20 to 50% of intentional injuries were reported to the police [8]. Similarly, in United States only 54% of such victims seeking emergency department (ED) care reported circumstances of events to police [9]. The discrepancies in official statistics for intentional injuries has almost never been assessed in LMICs most probably due to difficulties associated with data collection and linkages [3], [4].

Pakistan with the population of over 160 million inhabitants has a significant intentional injury disease burden [10], [11]. Official statistics showed that assaults resulted in 8.9 injuries per 100 000 inhabitants whereas homicide rate was 6.3 per 100 000 inhabitants [6]. A study in Rawalpindi division, Pakistan, comparing injury numbers reported in newspapers to the police statistics indicated differences in reporting of injury types in both sources during same period [12]. For instance, as compared to newspapers, police reported more non fatal road crashes, assaults, and homicides and fewer fatal crashes, deliberate self harm (DSH) and violence against women related injuries. However, newspapers based injury data is not reliable to assess discrepancies in official statistics [13]. Comparison of these statistics with other sources such as vital statistics, ambulance logs, or ED data could be useful to estimate such discrepancies for injury types in them [4]. Previously data linkage of police and ambulance log data were used to assess the completion of official statistics for road traffic injuries in Karachi, Pakistan [14]. Similar methods could be applied to intentional injuries for assessing discrepancies in police reporting, a poorly understood problem in Pakistan [15].

The objective of this study was to assess the differences in reporting of violence and DSH related injuries to police and ED in an urban town of Pakistan.

## Methods

### Ethics Statement

The study was approved by the institutional review board of the Rawalpindi Medical College and affiliated teaching hospitals. No written consent was taken from individuals reporting to police as such data are accessible to public. These data were collected with the permission of town police officer and were part of the usual police reporting. No specific data was collected for this research from those already reported to police. Similarly, ED data was recorded with verbal consent of the injured victim with hospital identification number, study method being approved as a part of trauma surveillance program by the institutional ethics review board.

### Study Setting and Design

Study setting was an urban town of Pakistan known as Rawalpindi city. Administratively it is known as Rawal town. It is situated in Northern Punjab near Islamabad, the capital city of Pakistan. In this study, incidences of violence and DSH related injuries and deaths were estimated from record linkage of police and ED data [14].

**Figure 1:**
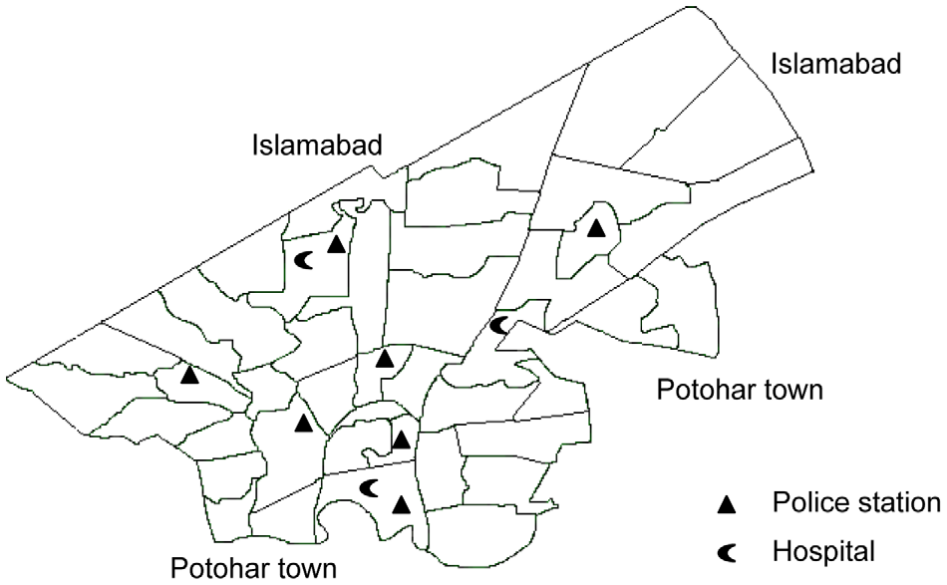
Map of Rawalpindi city consisting of 46 union councils. (Source: city district government web site).

In Rawalpindi city, law and order is maintained by seven police stations; New Town, Waris Khan, Banni, City, Pir Widhai, Ganj Mandi, and Sadiqabad police stations (figure 1). A register was maintained in each police station called as “Roznamcha” recording daily events reported to police. A first information report (FIR), a detailed account of the event, was registered when an offence was recognized under Pakistan Penal Code (PPC) and either of the interested parties wished to seek justice under those laws. These registers and reports were used to assess intentional injuries reported to police in this city.

Under Pakistani law, every DSH and intentional injury should be reported to designated local health facility known as Medico-legal centres (MLC) [15], [16]. In Rawalpindi city, three such centres exist in ED of teaching hospitals namely Holy Family Hospital, Benazir Bhutto Hospital (formerly called Rawalpindi General Hospital), and District Headquarter Hospital. A pilot injury surveillance program was established in the EDs of these hospitals from July 1, 2007 to June 30, 2008. Data collected from this ED injury surveillance was used for this study.

### Measures

Case was the person either injured or died as a result of interpersonal or collective violence or deliberate self-harm (DSH) and reported to a police station, ED, or both situated in Rawalpindi city from July 1, 2007 to June 30, 2008 [4]. In police station, intentional injuries were defined according to Criminal law (amendment) act of 1997 in Pakistan Penal Code (PPC) [16], [17]. Injury was defined as violence related if reported under section 332, 337, 337B, or 337C of PPC. Death was defined as violence related if reported under the section 300 and 315of PPC. Similarly, DSH related injury and death was defined if reported under section 325 of PPC. In ED, assessment of injury type was done according to the identified intent at the time of reporting injury [18]. Injury was defined as violence related if reported due to assault or due to operations of war or civil insurrections such as terrorism. Similarly, injury was defined as DSH if reported as a result of intentional self harm. In ED, death was counted when it occurred in the department.

Police stations were visited in February 2009 to obtain information on all prospectively reported violence and DSH related injuries and deaths over the period from July 1, 2007 to June 30, 2008. Police registers were searched by two investigators for above defined intentional injuries and FIR reports were consulted when available. Name, age, sex, injury type and mechanism, and outcome were recorded on a sheet for all victims.

ED dataset was obtained from an injury surveillance program which prospectively included all injury patients from ED in above mentioned hospitals from July 1, 2007 to June 30, 2008. ED data was collected by four hospital staff members round the clock coordinated by one surgery resident in each hospital. Injury details were collected on minimal dataset questionnaire including name, age and sex of victims, place, activity, nature, severity, and outcome of sustained injury. Injury intent was recorded according to World Health Organization's injury surveillance guidelines [18]. For violence related injuries, context and relationship of perpetrator were also registered [18]. Questionnaires were administered by face to face interview by data collectors after the initial management of these patients and obtaining informed consent. Police and ED data was coded on two separate Microsoft EXCEL spread sheets. Ten percent of the entries were double checked for errors.

### Analysis

In ED data, intentional injury victims that had occurred in Rawalpindi city were selected for further analysis. For violence related injuries and deaths, reported injury mechanism in ED was used to define whether it was blunt force, sharp cutting object, or firearm. Similar strategy was employed to regroup reported mechanism in police data. Frequencies of variables in the two datasets were computed. Observations in the two databases were matched by two investigators independently of each other on following criteria: date, time, name, age, and sex. Differences between two were resolved in the presence of a third investigator. Distributions of variables for matched observations were computed. Estimates ‘n’ of deaths and injuries with 95% confidence intervals (CI) limits were computed using following formula commonly known as two sample capture-recapture method [14], [19]:
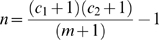


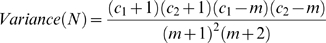


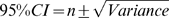
where ‘c_1_’ and ‘c_2_’ were the number of victims in ED and police data respectively and ‘m’ is the number of victims identified in both databases. Proportion of those matched with respect to police, ED and their aggregate for deaths and injuries were computed. In order to determine the completion of official statistics, ascertainment rate (observed/estimated count)*100 were computed for police, hospital, and their aggregate observations. Estimated counts with 95%CI were further used to compute violence and DSH related incidence of fatality and injury per 100,000 inhabitants with 95%CI for injury mechanism and type. Rawalpindi city population estimate of 2006 which was 1.6 million inhabitants was used to compute these rates [20], [21].

## Results

A total 1 016 intentional injury victims, mostly men (N = 945, 93.0%), reported their injury circumstance to seven police stations. Age of these victims was not recorded systematically. Seven of these victims' injuries resulted due to DSH. Similarly, 3 012 intentional injury victims, mostly men (N = 2 572, 85.4%), presented to ED in one year. One-tenth of injuries resulted from DSH (N = 331, 10.9%). Overall, 341 victims mostly men (N = 319, 93.5%) were found in both datasets accounting for 33.6% of police and 11.3% of ED records.

Most of the victims reporting to hospital were aged 16–45 years (N = 2 341, 77.3%). Adolescents (16–25 y) were most commonly involved in violence (N = 1 093, 41.9%) and DSH (N = 184, 56.4%) related injuries (Table 1 & 2). Common place for violence related injuries were markets and roads (N = 1 605, 59.9%) whereas most of DSH related injuries occurred at homes (N = 226, 68.3%). Almost three-fourth of violence related injuries (N = 233, 74.0%) and nearly all DSH related injuries (N = 120, 96.0%) in women occurred at homes. Most common group of perpetrators in case of men was friends and acquaintances (N = 1 120, 47.3%) whereas parents and close family members were commonest perpetrators (N = 139, 44.1%) in case of women. Blunt force (N = 1 310, 48.9%), sharp objects (N = 437, 16.3%), and firearms (N = 346, 12.9%) resulted in most of violence related injuries whereas poisoning (N = 171, 51.7%) was major injury mechanism in DSH related injuries. Nearly, one-sixth (N = 455, 16.9%) of violence related injuries resulted in concussion.

**Table 1:** Violence related injury victims presenting in emergency departments of teaching hospitals from Jul 07 to Jun 08.

	Total		Men		Women	
	N	(%)	N	(%)	N	(%)
Age (years)						
- ≤15	143	5.5	115	5.0	28	9.1
- 16–25	1 093	42.2	995	43.6	98	31.8
- 26–45	981	37.9	858	37.6	123	39.9
- >45	355	13.7	298	13.0	57	18.5
- Unknown	20	0.7	18	0.8	2	0.7
Place of injury						
- Market	818	30.5	805	34.0	13	4.1
- Road	787	29.4	743	31.4	44	14.0
- Home	769	28.7	536	22.7	233	74.0
- Work place	106	4.0	101	4.3	5	1.6
- Other	201	7.5	181	7.7	20	6.4
Context						
- Quarrel/assault	2 312	86.2	2 044	86.4	268	85.1
- Terrorism	108	4.0	97	4.1	11	3.5
- Gang activity	46	1.7	42	1.8	4	1.3
- Sexual assault	43	1.6	32	1.4	11	3.5
- Drug-related	23	0.9	20	0.9	3	1.0
- Others	149	5.6	131	5.5	18	5.7
Mechanism						
- Blunt force (club, stick, person)	1 310	48.9	1 162	49.1	148	48.9
- Sharp object (knife, cutting instrument)	437	16.3	394	16.6	43	16.3
- Firearm	304	11.3	264	11.2	40	11.3
- Fire, heat	12	0.5	12	0.2	7	0.2
- Others	618	23.0	618	22.9	77	22.9
Perpetrator						
- Friends/acquaintance	1 208	45.1	1 120	47.3	88	27.9
- Stranger/unknown	882	32.9	803	33.9	79	25.1
- Parents and relative	527	19.7	388	16.4	139	44.1
- Partner	64	2.4	55	2.3	9	2.9
Outcome						
- Treated and discharged	2 006	74.8	1 790	75.7	216	68.6
- Admitted/referred to hospital	588	21.9	503	21.3	85	27.0
- Died	24	0.9	21	0.9	3	0.9
- Others/unknown	63	2.4	52	2.2	11	3.5
Injury presentation						
- Cut/bite/open wound	1 024	38.2	922	39.0	102	32.4
- Organ system injury	483	18.0	434	18.3	49	15.6
- Concussion	452	16.9	395	16.7	57	18.1
- Fracture	147	5.5	125	5.3	22	7.0
- Bruise	91	3.4	81	3.4	10	3.2
- Sprain/strain	24	0.9	21	0.9	3	1.0
- Burn	22	0.8	15	0.6	7	2.2
- Other	438	16.3	373	15.8	65	20.6

**Table 2:** Deliberate self harm related injury victims presenting in emergency departments of teaching hospitals from Jul 07 to Jun 08.

	Total		Men		Women	
	N	(%)	N	(%)	N	(%)
Age (years)						
- ≤15	34	10.4	15	7.4	19	15.3
- 16–25	184	56.4	116	57.4	68	54.8
- 26–45	83	25.5	56	27.7	27	21.8
- >45	22	6.8	13	6.5	9	7.3
- Unknown	3	0.9	2	1.0	1	0.8
Place of injury						
- Home	226	68.3	106	51.5	120	96.0
- Roads	45	13.6	43	20.9	2	1.6
- Market	45	13.6	45	21.8	0	0.0
- Other	15	4.5	12	5.8	3	2.4
Mechanism						
- Poisoning	171	51.7	74	35.9	97	77.6
- Blunt force	25	7.5	18	8.7	7	5.6
- Sharp/cutting object	73	22.1	69	33.5	4	3.2
- Falls	16	4.8	12	5.8	4	3.2
- Firearm	9	2.7	8	3.9	1	0.8
- Fire	9	2.7	5	2.4	4	3.2
- Drowning	2	0.6	2	1.0	0	0.0
- Choking/hanging	1	0.3	1	0.5	0	0.0
- Others	25	7.5	17	8.3	8	6.4
Outcome						
- Treated and discharged	117	35.4	97	47.1	20	16.0
- Admitted/referred to hospital	194	58.6	95	46.1	99	79.2
- Died	8	2.4	7	3.4	1	0.8
- Others/unknown	12	3.6	7	3.4	5	4.0
Injury presentation						
- Cut/bite/open wound	65	19.6	59	28.6	6	4.8
- Fracture	12	3.6	8	3.9	4	3.2
- Organ system injury	27	8.2	19	9.2	8	6.4
- Burn	8	2.4	3	1.5	5	4.0
- Concussion	7	2.1	4	1.9	3	2.4
- Sprain/strain	2	0.6	1	0.5	1	0.8
- Bruise	3	0.9	3	1.5	0	0.0
- Other	207	62.5	109	52.9	98	78.4

For violence related deaths, 37.5% of aggregated records were matched; 42.8% of police and 75.0% of ED records (table 3). Estimated fatality count due to violence was 56 (95% CI: 46–64). Ascertainment rate was 85.7% for aggregated, 75.0% for police, and 42.8% for ED violence related death records. For violence related injuries 10.3% of aggregated records were matched; 33.2% for police and 12.1% for ED records. Estimated injury count due to violence was 7 990 (95% CI: 7 322–8 565). Ascertainment rate was 41.3% for aggregated, 12.1% for police; and 33.2% for ED violence related injury records. Violence related mortality rate was 3.5 (95% CI: 2.9–4.0) and injury rate was 499.3 (95% CI: 457.6–541.1) per 100 000 inhabitants.

**Table 3:** Incidences of deaths and injuries due to intentional injury type and mechanism in Rawalpindi city, (Jul 2007–Jun 2008).

				Records					Estimate	95% CI	Rate	95% CI
	Hospital		Police		Aggregate		Matched				(Per 100 000	inhabitants)
	N	(%)*	N	(%)*	N	(%)*	N	(%)**				
TYPE												
Violence												
-Death	24	(42.8)	42	(75.0)	48	(85.7)	18	(37.5)	56	[46–64]	3.5	[2.9–4.0]
-Injuries	2 657	(33.2)	967	(12.1)	3303	(41.3)	321	(10.3)	7 990	[7 322–8 656]	499.3	[457.6–541.1]
Deliberate self harm												
-Death	8	(47.1)	3	(17.6)	10	(58.8)	1	(10.0)	17	[4]–[30]	1.1	[0.3–1.9]
-Injuries	323	(39.9)	4	(0.5)	326	(40.3)	1	(0.3)	809	[101–1 516]	50.6	[6.3–94.8]
MECHANISM†												
Blunt												
-Death	7	(53.8)	8	(61.5)	11	(84.6)	4	(36.4)	13	[8]–[18]	0.8	[0.5–1.1]
-Injuries	1 303	(27.2)	726	(15.2)	1 832	(38.3)	197	(10.8)	4 788	[4 264–5 309]	299.2	[266.5–331.8]
Sharp												
-Death	1	(50.0)	2	(100.0)	2	(100.0)	1	(50.0)	2	‡	0.1	‡
-Injuries	436	(29.9)	109	(7.5)	513	(35.2)	32	(6.2)	1 456	[1 061–1 849]	91.0	[66.4–115.6]
Firearm												
-Death	14	(17.9)	20	(25.6)	31	(39.7)	3	(9.7)	78	[24–130]	4.9	[1.5–8.2]
-Injuries	290	(34.5)	51	(6.1)	324	(38.6)	17	(5.2)	840	[543–1 136]	52.5	[38.8–71.0]

*Ascertainment rate (observed/estimate*100).

**Proportion matched (matched/aggregate*100).

† Except deliberate self harm.

‡ Confidence intervals not estimated.

For DSH related deaths, 10.0% of aggregated records were matched; 33.3% of police and 12.5% of ED records. Estimated fatality count due to DSH was 17 (95% CI: 4–30). Ascertainment rate was 58.8% for aggregated, 17.6% for police, and 47.1% for ED DSH related death records. For DSH related injuries 0.3% of aggregated records were matched; 25.0% for police and 0.3% for ED records. Estimated injury count due to DSH was 809 (95% CI: 101–1 516). Ascertainment rate was 40.3% for aggregated, 0.5% for police, and 39.9% for ED DSH related injury records. DSH related mortality rate was 1.1 (95% CI: 0.3–1.9) and injury rate was 50.6 (95% CI: 6.3–94.8) per 100 000 inhabitants.

Firearms resulted in higher mortality rate per population as compared to other mechanisms (4.9 vs. ≤0.8 per 100 000 inhabitants). Most of the intentional injuries resulted from blunt mechanism (299.2 per 100 000 inhabitants).

## Discussion

In Rawalpindi city, using police or ED data alone in estimation of intentional injuries would be insufficient. Our results indicated that intentional injury deaths were reported more frequently than injuries alone. However, a comparison of injury types showed that DSH related injuries and deaths were far less reported to police than violence related injuries. Police records were more likely to be complete for violence related deaths as compared to injuries due to same mechanism. Firearms use appeared to be the major cause of violence related deaths.

This study indicated that police reported about twice as high violence related deaths than ED in Rawalpindi city. Opposing trends in reporting was found for such injuries as ED reported nearly three times more violence related injuries as compared to police. However, both deaths and injuries related to DSH were less likely reported to police as compared to ED. Only one out of 200 estimated DSH related injuries were reported to police. Such difference in reporting of injuries and deaths were previously documented for road traffic injuries where police data underestimated serious injuries by 21 times and under reported deaths by 44% [14]. It is possible that performance evaluation criteria set by higher police authorities where few police reported cases indicate better performance may facilitate this already identified malpractice of non-registering crime within Pakistan [22]. Although scientific evidence is poor in this regard but our study results indicated that police reporting policies for intentional injuries should be evaluated at least in this Pakistani city [14], [16], [23].

High under reporting of DSH related injuries in crime statistics has been highlighted in previous reports in Pakistan [15], [24], [25]. In fact, DSH is considered as an offence in Pakistani law with up to one year imprisonment and/or Rs. 10,000 fine [17]. In practice, although prosecution is rare yet extortion and harassment of families are frequent [15]. This may explain few or no police reporting of such events likely by the care givers in order to avoid adding further to the miseries of the bereaved victims and their families [24]. Moreover, DSH is considered as a sin in Islam, being the declared religion of 97% of Pakistanis [26]. This may lead to stigmatisation, avoidance of seeking professional help, and jeopardize reporting and development of control measures in healthcare setting [24], [26]. In Pakistan, DSH leads to significant disease burden and social consequences due to high youth involvement in such events [11]. Thoughtful consideration of these facts in Pakistani criminal law could be useful by avoiding criminalisation of this act, reducing stigma, and in improving reporting and tertiary prevention of these patients within Pakistani healthcare system, [11], [24], [26], [27].

Firearms were reported mechanism of ED reported intentional injury in slightly over one-tenth of victims. However, these resulted in significantly higher fatality rates than other injury mechanisms. Previous reports showed that firearms were the major injury mechanism in 61-78% of homicidal deaths in Pakistan [28]. Indeed, easy access to firearms in Pakistan lead to significant violence related injuries in Pakistan [29], [30]. These results strongly suggested that restricting firearm access could significantly decrease the violence related mortality in this city [4].

Domestic violence against women is highly prevalent in Pakistan [31]. These results indicated that women suffered most of the intentional injuries in their homes. Almost 34% of women reported physical abuse mostly by their partners in a Pakistani study [32]. A previous report showed that most common precipitating factors were daily conflicts, family related problems, disagreements between women and men on any decisions, choice preferences, and conflicts between two genders [31]. These results suggested that any domestic violence prevention program should focus on reaching women in their homes to prevent and control these injuries [4]. Such programs could be included in community based interventions already in place such as maternal and child health care in Pakistan [33].

There are several limitations to this study. Firstly, age and time were not used for matching purpose as decided earlier because age was not available in police records whereas reporting of time was not accurate in ED data. Further, as most police reports were not available we were not able to use more than one matching strategy to increase precision of our estimates [14]. Secondly, it was possible that the assumption that two datasets should be independent was not fulfilled as violence related injuries were often rushed to the hospitals with medico-legal officers and then cases might be referred to police stations. This type of bias increase the number of matches and underestimates the total number of injuries [34]. Nevertheless, these estimates clearly illustrated reporting differences of violence and DSH injuries in Rawalpindi city. Finally, the study results might under-estimate intentional injuries treated in private facilities [35]. Thus, these estimates should be considered as the bare minimum of such injuries in Rawalpindi, particularly those related to DSH [24].

Rawalpindi city, a predominantly urban town, is a strategic garrison city. From law enforcement and availability of healthcare facilities point of view, most of the population has adequate access to these services [21], [36]. This could result in better reporting of injuries to police as well as in ED rather than underreporting. As police reporting is same all over Pakistan, the under reporting of suicides in official statistics could easily be generalized for other middle-sized cities in Pakistan, most of them being situated in Punjab. Similarly, computed intentional injury rates could be considered as the minimum estimates of this disease burden in other middle sized cities of Pakistan.

Accurate assessment of intentional injuries is essential in every society. These results indicated that most of the victims were aged 16–25 years with women more involved in DSH and domestic violence as compared to men. These results suggested that use of police data could only be reliable for assessing violence related deaths and ED surveillance could be an alternate for assessing non fatal intentional injuries. A high underreporting of DSH related injuries to police could be explained by religious, social, and legal influences which lead to ‘criminalisation’ of DSH in Pakistan. This indicated that reporting of DSH related injuries and fatalities needs to be strengthened perhaps by analysing and modifying factors that lead to their underreporting in police stations. Better reporting of all intentional injuries perhaps at the level of EDs in coordination with properly staffed and equipped MLC could be useful for surveillance as well as implementing control measures for such type of injuries in Pakistan [15], [16].
